# Massive Exploration of Perturbed Conditions of the Blood Coagulation Cascade through GPU Parallelization

**DOI:** 10.1155/2014/863298

**Published:** 2014-06-16

**Authors:** Paolo Cazzaniga, Marco S. Nobile, Daniela Besozzi, Matteo Bellini, Giancarlo Mauri

**Affiliations:** ^1^Dipartimento di Scienze Umane e Sociali, Università degli Studi di Bergamo, 24129 Bergamo, Italy; ^2^SYSBIO Centre for Systems Biology, 20126 Milano, Italy; ^3^Istituto di Analisi dei Sistemi ed Informatica “Antonio Ruberti”, Consiglio Nazionale delle Ricerche, 00185 Roma, Italy; ^4^Università degli Studi di Milano-Bicocca, Dipartimento di Informatica, Sistemistica e Comunicazione, 20126 Milano, Italy; ^5^Università degli Studi di Milano, Dipartimento di Informatica, 20135 Milano, Italy

## Abstract

The introduction of general-purpose Graphics Processing Units (GPUs) is boosting scientific applications in Bioinformatics, Systems Biology, and Computational Biology. In these fields, the use of high-performance computing solutions is motivated by the need of performing large numbers of *in silico* analysis to study the behavior of biological systems in different conditions, which necessitate a computing power that usually overtakes the capability of standard desktop computers. In this work we present coagSODA, a CUDA-powered computational tool that was purposely developed for the analysis of a large mechanistic model of the blood coagulation cascade (BCC), defined according to both mass-action kinetics and Hill functions. coagSODA allows the execution of parallel simulations of the dynamics of the BCC by automatically deriving the system of ordinary differential equations and then exploiting the numerical integration algorithm LSODA. We present the biological results achieved with a massive exploration of perturbed conditions of the BCC, carried out with one-dimensional and bi-dimensional parameter sweep analysis, and show that GPU-accelerated parallel simulations of this model can increase the computational performances up to a 181× speedup compared to the corresponding sequential simulations.

## 1. Introduction

Mathematical modeling and computational analysis of biological systems nowadays represent an essential methodology, complementary to conventional experimental biology, to achieve an in-depth comprehension of the functioning of these complex systems [1, 2]. Given a model that describes the physical or logical interactions between the components of a biological system, different algorithms can be exploited to make predictions on the way this system behaves in both physiological and perturbed conditions. For instance, starting from distinct parameterizations of the model, simulation algorithms can be used to devise the different emergent behaviors that the system can present; the massive exploration of high-dimensional parameter spaces allows us to better understand the system functioning across a wide spectrum of natural conditions, as well as to derive statistically meaningful properties. Indeed, standard investigations of biological systems usually rely on computational methods that require the execution of a large number of simulations, such as parameter sweep analysis [3], sensitivity analysis [4], structure and parameter identifiability [5], parameter estimation [6–8], and reverse engineering of model topologies [9–13].

In this context, the use of general-purpose Graphics Processing Units (GPUs) has recently boosted many applications in scientific computing, where CPUs have traditionally been the standard workhorses. As a matter of fact, when several batches of simulations need to be executed, the necessary computing power can rapidly overtake the capabilities of standard desktop computers, therefore requiring high-performance computing solutions. After the introduction of general-purpose GPUs and of Compute Unified Device Architecture (CUDA, Nvidia's GPU programming language), the adoption of these graphics engines largely increased in research fields as Bioinformatics, Systems Biology, and Computational Biology (see an overview in [14–16]). Anyway, despite the remarkable advantages concerning the computational speedup, computing with GPUs usually requires the development and the implementation of* ad hoc* algorithms, since GPU-based programming substantially differs from CPU-based computing; as a consequence, scientific applications of GPUs might undergo the risk of remaining a niche for few specialists. To avoid such limitations, several packages and software tools have recently been released (see, e.g., [16–18]), so that also users with no knowledge of GPUs hardware and programming can access the high-performance computing power of graphics engines.

To investigate the dynamics of biological systems, either deterministic or stochastic approaches can be exploited [19], which are based on numerical integration (e.g., Euler's or Runge-Kutta methods [20]) or on Markov processes (e.g., Gillespie's algorithm [21]), respectively. To date, the most efficient algorithms to integrate a system of ordinary differential equations (ODEs), or to perform stochastic simulations of reaction-based models, are LSODA [22] and tau-leaping [23], respectively. In [24–26] we previously presented cupSODA and cuTauLeaping, the GPU-powered implementation of LSODA and tauleaping, respectively. cupSODA allows to run parallel deterministic simulations of a given mass-action based system of biochemical reactions, using the LSODA algorithm; cuTauLeaping represents a novel restructuring of the tau-leaping workflow that fits the GPU architecture and avoids any inefficiency drawback for coarse-grain massive parallel stochastic simulations.

In this work we introduce coagSODA, an extension of cupSODA that was specifically designed for the analysis of a model of the* blood coagulation cascade* (BCC). Blood is the subject of an intense scientific research, thanks to its key role in making diagnosis of numerous diseases [27]. Humans have evolved a complex hemostatic system that is able to maintain blood in a fluid state and allow the circulation through an intricate network of vessels; in particular, the presence of several fine-tuned feedback mechanisms in the BCC allows keeping all blood components within appropriate concentration ranges. The BCC consists in a complex network of cellular reactions which, under physiological conditions* in vivo*, are inhibited by the presence of intact endothelium [28]. Anyway, in response to any vascular injury, the hemostatic system is able to stop the blood leakage by rapidly sealing the defects in the vessels' wall [29].

In order to investigate the variations in blood coagulation components among individuals and to understand the corresponding response of the system to perturbed conditions, we consider here a computational perspective to study the BCC; we analyze the alterations (prolongation or reduction) of the time required to form the clot (i.e., the* clotting time*) by exploiting a reduced version of the mathematical model defined in [30]. This model describes the intrinsic, extrinsic, and common pathways of the BCC and, more importantly, it accounts for platelets activation, as well as the presence of several inhibitors (e.g., the Tissue Factor Pathway Inhibitor, antithrombin III, and C1-inhibitor) [31].

Numerous mathematical models of blood coagulation were developed in the last years, as they represent a useful tool for systematic studies of the intricate network of the coagulation cascade and allow obtaining a suitable reconstruction of empirical observations (see, e.g., [32–34]). The earliest models considered only simple steps of the whole BCC, such as the conversion of the clot fibrin by thrombin [35]; lately, Hockin et al. [36] developed a comprehensive ODE-based model of the extrinsic blood coagulation system. This model was then considered as reference by several research groups to investigate the thrombotic risk in healthy and ill populations [37, 38], or to understand other complex biochemical processes: for instance, in [39] the roles of protein C, protein S, and phospholipid surface actions were considered, while in [40] the influence of trace amounts of key coagulation proteases on thrombin generation was investigated. Recently, other works modeling the blood clotting process in a comprehensive manner have been published; besides the already mentioned model developed by Chatterjee et al. [30], we mention the model defined by Wajima et al. [32], which simulates the intrinsic, extrinsic, and common pathways, the vitamin K cycle, the therapy with the anticoagulant drugs warfarin and heparin, and the laboratory tests PT and aPTT, as well as the Taipan snake bite, which causes coagulopathy.

coagSODA, the GPU-accelerated simulator that we present and exploit in this work for the analysis of the BCC model, is a user-friendly and efficient tool that circumvents the need of manually defining the system of ODEs that describe the blood coagulation network. More precisely, coagSODA is able to automatically derive the system of (mass-action and Hill function-based) ODEs and then perform their numerical integration starting from the given set of 96 biochemical reactions, which fully describe the molecular interactions between all the species involved in the BCC* in vivo*. We show that coagSODA allows us to efficiently execute a large number of parallel deterministic simulations of the BCC at a considerable reduced computational cost with respect to CPUs. In particular, we exploit coagSODA to carry out one-dimensional and bi-dimensional parameter sweep analysis of the BCC, to the purpose of investigating the prolongation and the reduction of the clotting time in response to perturbed values of some reaction constants and of the initial concentration of some molecular species, chosen according to their meaning within the whole pathway.

The paper is structured as follows. In Section 2 we fully describe the mechanistic model of the BCC used in this work and present the simulation method at the basis of cupSODA tool to introduce the coagSODA simulator. In Section 3 we present the results obtained from the parameter sweep analysis of the BCC model, as well as a comparison of the computational performance of coagSODA with respect to a CPU-based implementation of LSODA. Finally, in Section 4 we conclude the paper with a discussion of the presented work and future research directions.

## 2. Materials and Methods

### 2.1. A Mechanistic Model of the Blood Coagulation Cascade

Blood is an essential component in human life, whose primary functions are to feed cells by delivering a multitude of nutrients, such as oxygen, and to carry away the cellular wastes, such as carbon dioxide. Specialized cells and fluids in blood perform many physiological functions and can be isolated and analyzed through specific laboratory tests, giving the opportunity to settle a person's health condition. All blood components are kept within appropriate concentration ranges by means of fine-tuned regulatory mechanisms, ruled by several feedback controls; the constancy of blood composition is maintained thanks to the circulation through an intricate network of vessels. In particular, humans evolved a complex hemostatic system that, under physiological conditions, maintains blood in a fluid state; however, in response to any vascular injury, this system is able to rapidly react and seal the defects in the vessels' wall in order to stop the blood leakage [29]. Indeed, the circulatory system is self-sealing; otherwise, a continuous blood flow from even the smallest wound would become a threat for the individual's life.

To allow blood coagulation, in humans there exist 13 blood clotting proteins, called* coagulation factors*, which are usually designated by Roman numerals I through XIII. As a consequence of a vascular injury, platelets become active and the Tissue Factor (TF, also called factor III) is exposed in the subendothelial tissue, starting the* blood coagulation cascade* (BCC). The ultimate goal of the BCC is to convert prothrombin (factor II) into thrombin (factor IIa—i.e., the active factor II), the enzyme that catalyzes the formation of a clot. Traditionally, the BCC is divided into the* extrinsic* and* intrinsic pathways*, both leading to the activation of factor X [41]. The last part of the cascade, downstream of this factor, is called the* common pathway* and leads to the formation of fibrin monomers, whose polymers finally constitute the backbone of the clot.

Excluding thrombin, all the enzymes involved in blood clotting are characterized by a low activity, which increases upon binding to a specific protein cofactor (e.g., factors V and VIII) or to appropriate phospholipid surfaces (e.g., the plasma membranes of active platelets) [41]. Even calcium ions have a central role in coagulation, since they are essential to start and enhance numerous reactions; without calcium ions the blood coagulation cannot occur [42]. In the BCC pathways, the activity of the various active proteases is limited by several inhibitory factors, which allow regulating the whole cascade. When the hemostatic system is unregulated, thrombosis (i.e., the formation of a blood clot obstructing the blood flow in vessels) may occur due to impairment in the inhibitory pathway, or because the functioning of the natural anticoagulant processes is overwhelmed by the strength of the hemostatic stimulus [29].

The BCC model we consider in this work is a slightly reduced version of the “Platelet-Plasma” deterministic model defined in [30], built upon a previous model [36], which describes all parts of blood coagulation: the platelets activation and aggregation; the extrinsic, intrinsic and common pathways (with the exception of factor XIII); the action of several inhibitory molecules (Tissue Factor Pathway Inhibitor, antithrombin III, C1-inhibitor, *α*1-antitrypsin, and *α*2-antiplasmin). In addition, to simulate the coagulation process* in vitro*, Chatterjee et al. also modeled the action of corn trypsin inhibitor (CTI), which inhibits the activation of the so-called contact system, as well as the action of the fluorogenic substrate Boc-VPR-MCA widely used in laboratories for thrombin titration [30]. The role of calcium was not explicitly included in the model but considered as a nonlimiting factor, as in living organisms this ion very rarely drops to such low levels able to alter the kinetics of the formation of the fibrin clot [43].

Since the aim of this work is the investigation of blood coagulation* in vivo*, we exclude a small set of reactions given in [30] that have no effect on the clotting time. To be more precise, we do not consider the reactions occurring* in vitro* (namely, entries 28 and 35 in Table  1 in [30]), as well as the reactions downstream the fibrinogen conversion, that is, the interactions between the fibrin polymers, thrombin, and antithrombin III (namely, entries 55, 56, and 57 in Table  1 in [30]).

**Figure 1:**
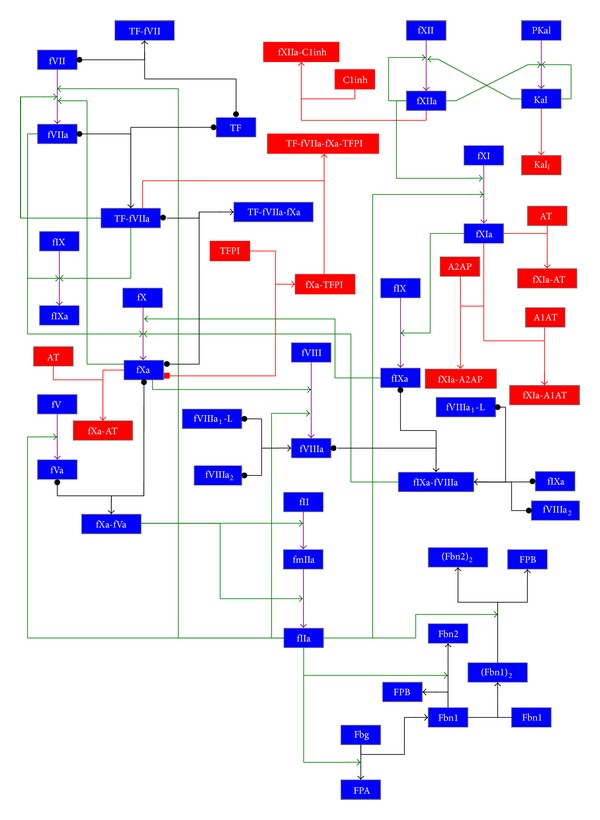
Graphical representation of the blood coagulation cascade model considered in this work.* Legend*. Blue box: coagulation factor; red box: inhibitor and related complexes. Black arrow: complex formation; green arrow: catalytic activation; violet arrow: activation; red arrow: inhibition. The reaction is reversible if the arrow tail consists in a dot.

For the sake of completeness, we describe in Table 1 the BCC model considered in this work, overall consisting in 96 reactions among 71 molecular species. A graphical sketch of the main molecular interactions among the BCC components is given in Figure 1.

The model can be partitioned into four functional modules.

1. The first module corresponds to the* extrinsic pathway*, which consists in
  - the formation of a complex between Tissue Factor and factor VII (modeled by reactions *r*_1_,…, *r*_4_);
  - the activation of factor VII by the complex between Tissue Factor and factor VIIa (reaction *r*_5_);
  - the activation of factor IX by the complex between Tissue Factor and factor VIIa (reactions *r*_13_, *r*_14_, *r*_15_) and by factor VIIa (reactions *r*_78_, *r*_79_, *r*_80_);
  - the activation of factor X by the complex between Tissue Factor and factor VIIa (*r*_8_,…, *r*_12_) and by factor VIIa (*r*_81_, *r*_82_, *r*_83_).
2. The second module corresponds to the* intrinsic pathway*, which consists in
  - the formation of a complex between factor VIIIa and factor IXa (modeled by reactions *r*_18_ and *r*_19_);
  - the activation of factor X by the complex between factor VIIIa and factor IXa (reactions *r*_20_, *r*_21_, *r*_22_) and by factor IXa (reactions *r*_72_, *r*_73_, *r*_74_);
  - the activation of factor XII by factor XII itself (reaction *r*_44_), by factor XIIa (reactions *r*_45_, *r*_46_, *r*_47_), and by kallikrein (reactions *r*_51_, *r*_52_, *r*_53_);
  - the activation of prekallikrein by factor XIIa (reactions *r*_48_, *r*_49_, *r*_50_) and by kallikrein (reaction *r*_54_);
  - the activation of factor XI by factor XIIa (reactions *r*_61_, *r*_62_, *r*_63_) and by factor XIa (reaction *r*_64_);
  - the activation of factor IX by factor XIa (reactions *r*_69_, *r*_70_, *r*_71_);
  - the dissociation of free factor VIIIa (reactions *r*_23_ and *r*_24_);
  - the dissociation of factor VIIIa in complex with other factors (reactions *r*_25_ and *r*_26_).
3. The third module corresponds to the* common pathway*, which consists in
  - the activation of factor II by factor Xa (modeled by reaction *r*_16_) and by the complex between factor Xa and factor Va, through the formation of the intermediate meizothrombin (reactions *r*_30_,…, *r*_33_);
  - the activation of factor VII by factor Xa (reaction *r*_6_) and by factor IIa (reaction *r*_7_);
  - the activation of factor VIII by factor Xa (reactions *r*_75_, *r*_76_, *r*_77_) and by factor IIa (reaction *r*_17_);
  - the formation of a complex between factor Xa and factor Va (reactions *r*_28_ and *r*_29_);
  - the activation of fibrinogen by factor IIa (reactions *r*_84_,…, *r*_96_);
  - the activation of factor V by factor IIa (reaction *r*_27_);
  - the activation of factor XI by factor IIa (reactions *r*_58_, *r*_59_, *r*_60_).
4. The fourth module describes the* inhibition of coagulation*, carried out through the main inhibitors of the BCC (antithrombin, Tissue Factor Pathway Inhibitor, C1-inihibitor, *α*1-antitrypsin, and *α*2-antiplasmin). These are tight binding inhibitors belonging to the serpin superfamily, which form irreversible complexes. This module consists in
  - the inhibition of factor Xa by Tissue Factor Pathway Inhibitor (modeled by reactions *r*_34_ and *r*_35_);
  - the inhibition of the complex between Tissue Factor, factor VIIa, and factor Xa by Tissue Factor Pathway Inhibitor (reactions *r*_36_, *r*_37_, *r*_38_);
  - the inhibition of factor IIa by antithrombin (reactions *r*_40_ and *r*_42_);
  - the inhibition of factor Xa by antithrombin (reaction *r*_39_);
  - the inhibition of factor IXa by antithrombin (reaction *r*_41_);
  - the inhibition of factor XIa by antithrombin (reaction *r*_65_) and by C1-inhibitor (reaction *r*_66_);
  - the inhibition of factor XIIa by C1-inhibitor (reaction *r*_56_), by antithrombin (reaction *r*_57_), by *α*1-antitrypsin (reaction *r*_67_), and by *α*2-antiplasmin (reaction *r*_68_);
  - the inhibition of the complex between Tissue Factor and factor VIIa by antithrombin (reaction *r*_43_);
  - the inhibition of kallikrein (reaction *r*_55_).

The values of the initial concentrations of the molecular species occurring in the BCC model are given in Table 2. According to [30], the concentrations of complexes and active factors were set to 0, except for the active factor VII, which is physiologically present in the blood circulation, even in the absence of damage, in a concentration that is approximately equal to 1% of the corresponding inactive factor [42].

The system of ordinary differential equations (ODEs), needed to carry out the simulations and the parameter sweep analysis presented in Section 3, was derived from the reactions given in Table 1 according to the* mass-action law*, with the exception of 14 reactions belonging to the set *S*_*ɛ*_ = {*r*_19_, *r*_21_, *r*_23_, *r*_29_, *r*_31_, *r*_35_, *r*_46_, *r*_49_, *r*_52_, *r*_62_, *r*_70_, *r*_73_, *r*_76_, *r*_82_}. The mass-action law is the fundamental empirical law that governs biochemical reaction rates which states that, in a dilute solution, the rate of an elementary reaction is proportional to the product of the concentration of its reactants raised to the power of the corresponding stoichiometric coefficient [44]. The reactions in *S*_*ɛ*_ are instead influenced by a specifically defined variable *ε*, depending on a Hill function, fit against experimental data which quantify the platelet activation status, which is used to model the physiological levels of thrombin concentration as a function of platelet activation, as thoroughly described in [30]. More precisely, the value of *ε* influences the formation of some complexes occurring on the platelets' surface, by modifying the activity of reactions of the form

(1) A+B⇌k2k1AB.

Namely, *ε* intervenes with the dissociation constant of these reactions, so that the corresponding standard ODEs are changed to yield new equations of the form

(2) $$\begin{array}{c} \frac{d\left[AB\right]}{dt}=k_{1}\cdot \left[A\right]\cdot \left[B\right]-\frac{k_{2}\cdot \left[A\right]\cdot \left[B\right]}{ɛ}. \end{array}$$

The value of *ε* in (2) depends on the following Hill function *H*, which quantifies the state of platelets activation according to the thrombin concentration (here denoted as [fIIa]), that is, the factor catalyzing the formation of the fibrin clot:

(3) $$\begin{array}{c} H\left(\left[\text{fII}{\text{a}}^{*}\left(t\right)\right]\right)=\frac{{\left[\text{fII}{\text{a}}^{*}\left(t\right)\right]}^{1.6123}}{{\left[\text{fII}{\text{a}}^{*}\left(t\right)\right]}^{1.6123}+{\left(2.4279\cdot 10^{-9}\right)}^{1.6123}}, \end{array}$$

where [fIIa*(*t*)] = max⁡_*t*′∈[0,*t*]_{[fIIa(*t*′)]}, for a chosen time interval [0, *t*] of simulation.

**Figure 2:**
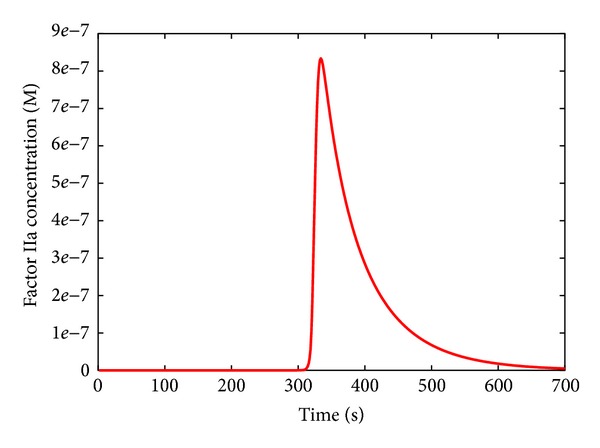
Dynamics of thrombin (factor IIa) in physiological condition.

The value [fIIa*(*t*)] represents the maximum transient thrombin concentration and is needed to simulate the fact that, in physiological conditions, the thrombin concentration starts decreasing after rising to a peak (Figure 2). So doing, [fIIa*(*t*)] never decreases once it reaches its maximum magnitude; [fIIa*(*t*)] is equivalent to [fIIa(*t*)] until the concentration of factor IIa reaches the peak, while thereafter it remains constant at that value, which is the maximum in the considered time interval [0, *t*]. Function *H* allows simulating the physiological condition, whereby platelets remain active also when the thrombin concentration decreases.

For a given concentration of factor IIa, the maximum platelets activation state *ε*_max⁡_ is defined as

(4) $$\begin{array}{c} \varepsilon_{\max}=\varepsilon_{\text{ma}{\text{x}}_{0}}+\left(1-\varepsilon_{\text{ma}{\text{x}}_{0}}\right)\cdot H\left(\left[\text{fII}{\text{a}}^{*}\left(t\right)\right]\right), \end{array}$$

where *ε*_max_0__ defines the basal activation state of the platelets at simulation time *t* = 0. The value *ε*_max_0__ is initially set to 0.01, assuming a basal 1% binding strength of coagulation factors to the resting platelets' surface. When the full activation of platelets is reached, *ε*_max⁡_ is equal to 1 and the complex dissociation constants are minimized (see [30] for more details).

### 2.2. CUDA Architecture and the coagSODA Simulator

Introduced by Nvidia in 2006, Compute Unified Device Architecture (CUDA) is a parallel computing platform and programming model that provides programmers with a framework to exploit GPUs in general-purpose computational tasks (GPGPU computing). GPGPU computing is a low-cost and energy-wise alternative to the traditional high-performance computing infrastructures (e.g., clusters of machines), which gives access to the tera-scale computing on common workstations of mid-range price. However, due to the innovative architecture and the intrinsic limitations of GPUs, a direct porting of sequential code on the GPU is most of the times unfeasible, therefore making it challenging to fully exploit its computational power and massive parallelism [45].

**Figure 3:**
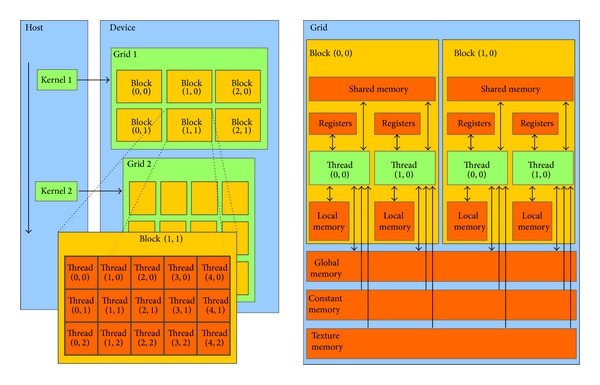
Schematic description of CUDA's architecture, in terms of threads and memory hierarchy.* Left Side*. Threads organization: a single kernel is launched from the host (the CPU) and is executed in multiple threads on the device (the GPU). Threads can be organized in three-dimensional structures named blocks which can be, in turn, organized in three-dimensional grids. The dimensions of blocks and grids are explicitly defined by the programmer.* Right Side*. Memory hierarchy: threads can access data from many different memories with different scopes. Registers and local memories are private for each thread. Shared memory lets threads belonging to the same block communicate and has low access latency. All threads can access the global memory, which suffers high latencies, but it is cached since the introduction of the Fermi architecture. Texture and constant memory can be read from any thread and are equipped with a cache as well; in this work we exploit the constant memory. Figures are taken from Nvidia's CUDA programming guide [60].

CUDA combines the single instruction multiple data (SIMD) architecture with multithreading, which automatically handles the conditional divergence between threads. The drawback of such flexibility is that any divergence of the execution flow between threads causes a serialization of the execution, which affects the overall performances. Under CUDA's naming conventions, the programmer implements the* kernel*, that is, a C/C++ function, which is loaded from the host (the CPU) to the devices (one or more GPUs) and replicated in many copies named* threads*. Threads can be organized in three-dimensional structures named* blocks* which, in turn, are contained in three-dimensional* grids* (a schematic description is given in Figure 3, left side). Whenever the host runs a kernel, the GPU creates the corresponding grid and automatically schedules each block on one free streaming multiprocessor available on the GPU, allowing a transparent scaling of performances on different devices. Threads within a block are executed in groups of 32 threads named* warps*.

**Figure 4:**
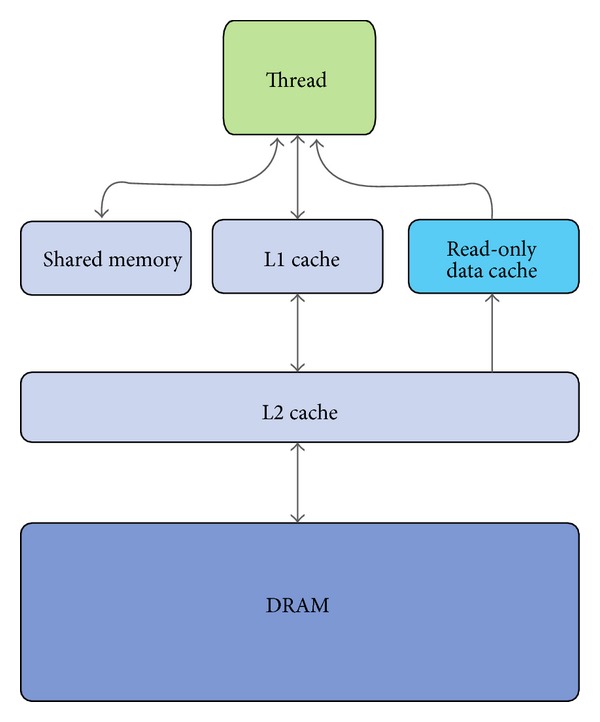
Schematic description of memory hierarchies in Fermi and Kepler architectures. GPUs relying on these architectures are equipped with a two-level data cache and a read-only data cache. Shared memory and L1 cache share the same on-chip 64 KB memory banks; the amount of memory can be reconfigured by the user, according to the specific needs of the application. Figure taken from Nvidia's Kepler GK110 whitepaper [57].

The GPU is equipped with different kinds of memory. In this work, we exploit the* global memory* (accessible from all threads), the* shared memory* (accessible from threads of the same block), the* local memory* (registers and arrays, accessible from owner thread), and the* constant memory* (cached and not modifiable). A schematic representation of this memory hierarchy is shown in Figure 3, right side. To achieve the best performances, the shared memory should be exploited as much as possible; however, it is very limited (i.e., 49152 bytes for each multiprocessor, since the introduction of the Fermi architecture) and it introduces restrictions on the blocks' size. On the other hand, the global memory is very large (thousands of MBs) but suffers from high latencies. A solution to this problem was implemented on the Fermi architecture, where the global memory is equipped with a L2 cache. This architecture also introduced the possibility to balance 64 KB of fast on-chip memory between the shared memory and L1 cache using two possible configurations, 48 KB for the shared memory and 16 KB for L1 cache, or 16 KB for the shared memory and 48 for L1 cache. The Kepler architecture, used in this paper, allows a third and perfectly balanced configuration, where shared memory and L1 cache obtain the same amount of memory (32 KB). See Figure 4 for a schematization of the memory architecture.

The systematic analysis of models of biological systems often consists in the execution of large batches of simulations. One of the standard analyses that can be executed on such kind of models regards an intensive search within the parameters space, which requires large numbers of independent simulations. In order to reduce the computational burden, we previously implemented on the CUDA architecture one of the most efficient numerical integration algorithms for ODEs, LSODA, that is able to automatically recognize stiff and nonstiff systems and to dynamically select between the most appropriate integration procedure (i.e., Adams method in the absence of stiffness and the Backward Differentiation Formulae otherwise) [22]. This GPU-powered tool is called cupSODA [24, 25] and exploits CUDA's massive parallelism to execute different and independent simulations in each thread, thus reducing the computational time required by a standard CPU counterpart of LSODA.

Besides being very efficient for the simulation of many independent simulations, cupSODA is also user-friendly. LSODA was originally designed to solve ODEs systems written in the canonical form, but the user is supposed to specify the system of ODEs by implementing a custom C function that is passed to the algorithm; moreover, in order to speed up the computation when dealing with stiff systems, in LSODA the Jacobian matrix associated with the ODEs system must be implemented as a custom C function as well. On the contrary, cupSODA was conceived as a* black-box* simulator that can be easily used without any programming skills. cupSODA consists in a tool to automatically convert a generic mechanistic reaction-based model of a biological system into the corresponding set of ODEs, to comply with the mass-action kinetics [46], and to directly encode the obtained system, along with the corresponding Jacobian matrix, as C arrays.

This fully automatic methodology can be exploited for the simulation of models whose chemical kinetics is based on the mass-action law. The BCC model described in Section 2.1, though, includes a set *S*_*ε*_ of reactions that do not follow the mass-action kinetics [30]; the platelets activity is not explicitly modeled by biochemical reactions, but it is realized by modulating the rate of the dissociation of the complexes formed on a platelet's surface by means of the variable *ε*, which is calculated with a special equation during the integration steps. For this reason, cupSODA was used as a starting point for the development of a new tool able to compute at* run-time* the specific kinetics of this set of reactions.

This new CUDA-powered simulation tool, named coagSODA, is specifically tailored for the simulation of the BCC model developed in [30]. In particular, coagSODA realizes the run-time calculation of the *ε* value required to correctly simulate the activity of platelets, which is determined according to the equations described in Section 2.1. The platelets activation state *ε* is calculated at each time instant by solving the following differential equation:

(5) $$\begin{array}{c} \frac{dɛ}{dt}=k\left(\varepsilon_{\max}-\varepsilon \right), \end{array}$$

where the constant *k* is inversely proportional to the time scale of platelets activation and is set to 0.005. This is consistent with the fact that platelets do not instantly achieve their maximum attainable activation state (*ε*_max⁡_), but they reach it on a physiologically relevant timescale [30].

Dealing with the 14 reactions in the set *S*_*ε*_ that are influenced by the Hill function *H* (see Section 2.1), the value of [fIIa*(*t*)] must be stored on the GPU because, during each integration step, coagSODA recalculates (4) which exploits the value [fIIa*(*t*)] to determine a new *ε* value. Since CUDA's architecture does not offer static variables, the information for each thread has to be memorized in the global memory. The accesses to the global memory and the computational costs due to these additional calculations slow down the integration process, with respect to the integration of ODEs performed according to strictly mass-action based kinetics (as in the cupSODA simulator). Nevertheless, in Section 3.3 we show that our parallel implementation largely outperforms a sequential counterpart of the LSODA algorithm.

As in the case of cupSODA [24, 25], coagSODA exploits the shared memory to improve performances by storing the current state and time of the simulations in these low-latency memory banks. Despite the improvement of performances ensured by this solution, it strongly affects the occupancy of GPU's multiprocessors and therefore it represents the limiting factor for the number of blocks that can be executed simultaneously; as a matter of fact, coagSODA is limited to 2 blocks per streaming multiprocessor on GPUs based on the Fermi architecture, with a reduced exploitation of the GPU with respect to the theoretical 8 blocks allowed by this architecture.

## 3. Results and Discussion

In this section we discuss the results of the parameter sweep analysis (PSA) carried out on the BCC model, to the aim of investigating either the prolongation or the reduction of the clotting time in response to perturbed values of some reaction constants and of the initial concentration of some molecular species, chosen according to their meaning within the whole pathway. PSA was performed by generating a set of different initial conditions, corresponding to different parameterizations of the model, and then automatically executing the deterministic simulations with coagSODA. The use of GPU technology is fundamental in this type of analysis, especially for large biological systems as the BCC is, because it drastically reduces the computational time.

The sweep analysis for single parameters (PSA-1D) was performed considering a logarithmic sampling of numerical values of each parameter under investigation (reaction constant or initial molecular concentration) within a specified range with respect to its physiological reference value. The sweep analysis over pairs of parameters (PSA-2D) was performed by simultaneously varying the values of two parameters within a specified range, considering a logarithmic sampling on the resulting lattice. The logarithmic sampling allows uniformly spanning different orders of magnitude of the parameters value using a reduced and fine-grained set of samples, therefore efficiently analyzing the response of the system in a broad range of conditions.

**Figure 5:**
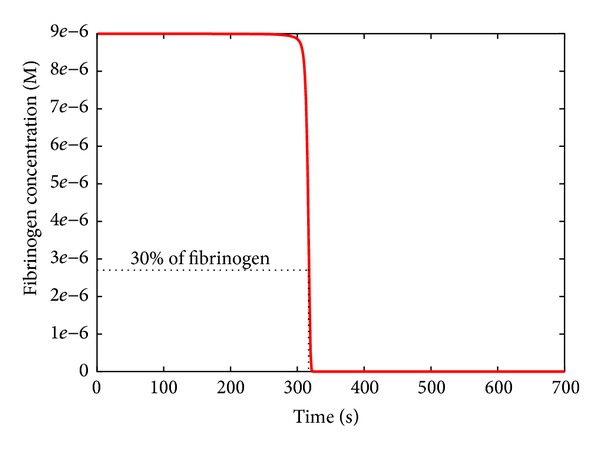
Dynamics of fibrinogen in physiological condition. The clotting time is defined as the time necessary to convert the 70% of the fibrinogen into fibrin.

To determine the response of the BCC to perturbed conditions, we chose the* clotting time* (CT) as output of the PSA. The CT is defined as the time necessary to convert the 70% of the fibrinogen into fibrin (see Figure 5), conventionally assumed to correspond to the time required to form the clot [47], and it is generally used in laboratory tests for monitoring the therapy with anticoagulant drugs. According to the model defined in [30], the reference value of CT is around 300 seconds in physiological conditions. We investigate here the response of the BCC by evaluating the CT in various conditions, corresponding to different values of the reaction constants, varying over six orders of magnitude with respect to their physiological values (i.e., three below and three above the reference values, if not otherwise specified), as well as to different values of the initial molecular concentrations, and varying over twelve orders of magnitude with respect to their physiological values (i.e., six below and six above the reference values, if not otherwise specified).

The total number of parallel simulations executed to carry out these analyses was 100 for PSA-1D over reaction constants, 200 for PSA-1D over initial concentrations, and 1600 for PSA-2D.

Finally, we present the comparison of the performance of the CPU and GPU to run an increasing number of simulations of the BCC model, to prove the efficiency of coagSODA.

### 3.1. PSA-1D of the BCC Model

#### 3.1.1. Reaction *r*_44_

The first PSA was performed to determine the value of the kinetic constant for the autoactivation of factor XII (reaction *r*_44_ in Table 1), which corresponds to an upstream process in the intrinsic pathway. This analysis was motivated by two considerations. Firstly, by using the full parameterization given in [30], the action of the intrinsic pathway turns out to be fundamental for the BCC* in vivo*, which is in contrast to experimental observations which indicate that the extrinsic pathway is the main responsible of clot formation. Secondly, in [30] all constants values have a reference to experimental measurements, except for the constant of this reaction (which corresponds to entry 29 in Table 1 in [30]).

**Figure 6:**
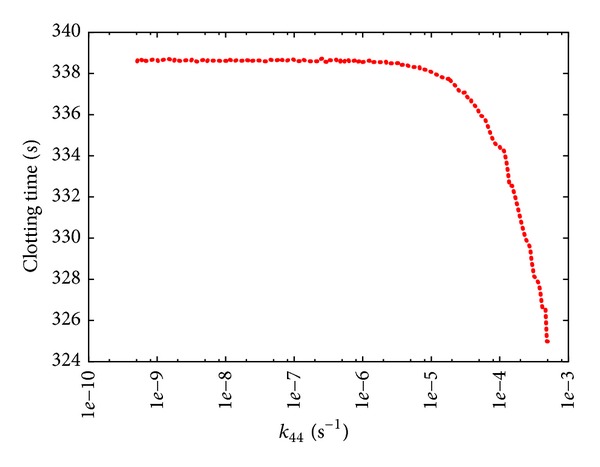
Clotting time according to PSA-1D over constant *k*_44_ of reaction *r*_44_: XII → XIIa. The reference value of *k*_44_ used in [30] is 5 · 10^−4^s^−1^; the sweep range is [5 · 10^−10^, 5 · 10^−4^].

Figure 6 shows the results of this PSA-1D, where *k*_44_ was varied over six orders of magnitude, considering the value given in [30] as the upper limit of the sweep interval. We can observe that, by decreasing the value of *k*_44_ with respect to the value considered in [30], the CT increases with respect to its reference value; however, for values of this constant lower than 1.00 · 10^−6^ s^−1^ the CT remains unaltered, and this can be intuitively explained by the fact that in this situation the fibrinogen is mainly activated by the extrinsic pathway.

Consequently, we assigned the value 7.00 · 10^−6^ s^−1^ to *k*_44_, achieving a CT that is comparable to the experimental observations of the BCC* in vivo*. This new value was used in all PSA discussed in what follows.

#### 3.1.2. Reactions *r*_27_ and *r*_58_

In the next PSA we investigated the effect of the perturbation of the kinetics of two pivotal reactions of the BCC model. Reaction *r*_27_, which describes the catalytic activation of factor V by factor IIa, was chosen because it represents the main positive feedback within the common pathway; reaction *r*_58_, which is involved in the activation of factor XI by binding to factor IIa, was chosen because it represents the main positive feedback in the intrinsic pathway (Figure 1). Moreover, preliminary PSA over all reaction constants of the BCC model given in Table 1 evidenced that these two reactions are among the most relevant steps of the coagulation network.

**Figure 7:**
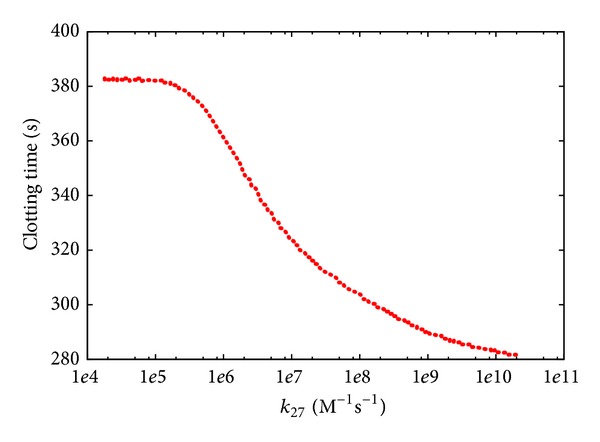
Clotting time according to PSA-1D over constant *k*_27_ of reaction *r*_27_: IIa + V → IIa + Va. The reference value is 2 · 10^7^ M^−1^  s^−1^; the sweep range is [2 · 10^4^, 2 · 10^10^].

**Figure 8:**
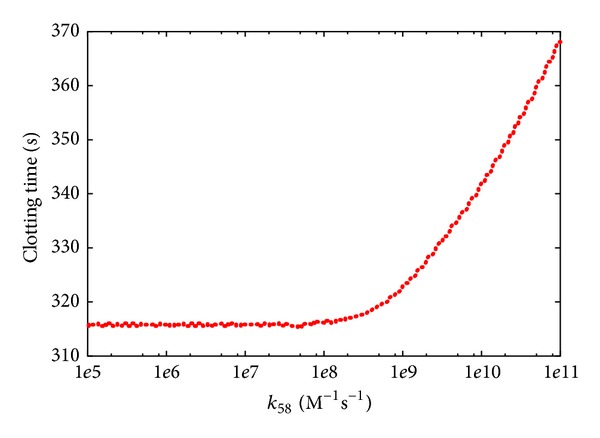
Clotting time according to PSA-1D over constant *k*_58_ of reaction *r*_58_: IIa + XI → IIa-XI. The reference value is 1 · 10^8^ M^−1^  s^−1^; the sweep range is [1 · 10^5^, 2 · 10^11^].

The PSA-1D over *k*_27_ (Figure 7) shows that the CT is very sensitive to the perturbation of the rate of this reaction, when the reference value of its constant (i.e., *k*_27_ = 2 · 10^7^ M^−1^ s^−1^) is either increased or decreased; in particular, when *k*_27_ is very low, a plateau in CT is reached since the strength of the positive feedback exerted by factor IIa is largely reduced, a condition where the contribution of the amplification of the hemostatic stimulus (due by the common pathway) to the formation of the clot is basically not effective. On the other side, the PSA-1D over *k*_58_ (Figure 8) shows that, while a decrease of the constant with respect to its reference value (i.e., *k*_58_ = 1 · 10^8^ M^−1^ s^−1^) does not have any substantial effect, an increase of its value leads to a progressive increase in the CT. This increase is due to the fact that factor IIa is sequestered in the formation of a complex with factor XI, and hence it is no longer available as a free component in blood to participate in other reactions, especially those reactions of the extrinsic pathway which principally lead to the clot formation* in vivo*. This behavior highlights that the intrinsic pathway has a secondary role in blood coagulation* in vivo*, compared with the extrinsic pathway, as also evidenced by various experimental observations [48].

#### 3.1.3. Factors VIII, IX, and II

The next set of PSA-1D was realized by varying the initial concentrations of factors VIII, IX, and II. These factors were selected since both an excess and a deficiency of their concentrations lead to diseases related to blood clotting.

**Figure 9:**
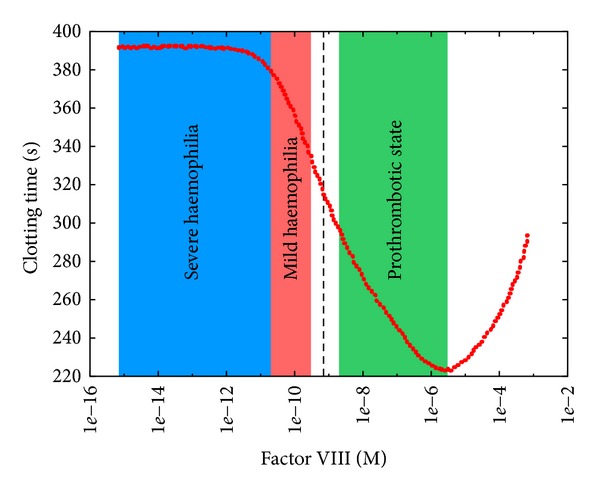
Clotting time at different initial concentrations of factor VIII. The reference value is 7.00 · 10^−10^ M (dashed black line); the sweep range is [7.00 · 10^−16^, 7.00 · 10^−4^]. The blue area indicates a condition in which factor VIII concentration is less than 1% of its physiological concentration, corresponding to a situation of severe haemophilia; the red area indicates a condition in which factor VIII concentration is between 1% and 30% of its physiological concentration, corresponding to a situation of mild haemophilia; the green area indicates a condition in which factor VIII concentration is greater than 130% of its physiological concentration, corresponding to a situation of hypercoagulability.

The PSA-1D over factor VIII (Figure 9) shows that increasing the initial concentration of this factor results in decreasing the CT, suggesting the possible presence of hypercoagulable states in these perturbed conditions. As a matter of fact, high levels of factor VIII cause an increased risk of deep vein thrombosis and pulmonary embolism [49]. On the other hand, individuals with less than 1% of the average concentration of factor VIII show a severe haemophilia A, characterized by higher CT (Figure 9), and require infusions of plasma containing the deficient factor; otherwise, frequent spontaneous bleeding would occur [50]. When the concentration of this factor is between 5% and 30% of the average concentration, individuals still risk bleeding in case of trauma [50].

**Figure 10:**
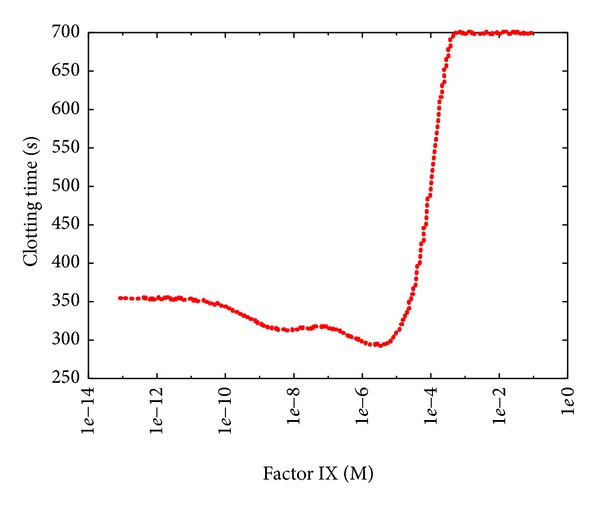
Clotting time at different initial concentrations of factor IX. The reference value is 9.00 · 10^−8^ M; the sweep range is [9.00 · 10^−14^, 9.00 · 10^−2^].

The PSA-1D over factor IX (Figure 10) shows only a slight decrease of the CT as the initial concentration of fIX increases; this is in contrast to recent studies that demonstrated how the excess of factor IX leads to an increased risk of deep vein thrombosis [51]. This result can be explained by considering that (i) a high concentration of factor IX is not sufficient to bring about coagulation problems, though when the concentration of other factors is above the average value (yet not at pathological levels), prothrombotic states can be observed; (ii) in this model we consider average values as initial concentrations of factors; however, individuals are characterized by different (balanced) combinations of procoagulant and anticoagulant factor levels that altogether contribute to define a unique coagulation phenotype that reflects the developmental, environmental, genetic, nutritional, and pharmacological influences of each individual [52]. On the contrary, the lack of factor IX causes haemophilia B, characterized by higher CT with respect to the reference value (Figure 10).

Furthermore, by comparing the PSA-1D of factors VIII and IX (Figures 9 and 10) it is clear that haemophilia A is more serious than haemophilia B, since the CT achieved in conditions of factor VIII deficiency is higher than the CT obtained in the case of factor IX deficiency.

In both PSA-1D over factors VIII and IX we observed, after the initial decrease of the CT, an unexpected increase of the CT as the factor concentration increases. This counterintuitive behavior arises at very high concentrations of these factors (with respect to the average physiological levels) and, to the best of our knowledge, it was never observed* in vivo*. Nonetheless, it would be interesting to verify, by means of* ad hoc* laboratory experiments, if the model correctly describes the behavior of the BCC even in these conditions or, on the contrary, it is not predictive in these extreme situations.

**Figure 11:**
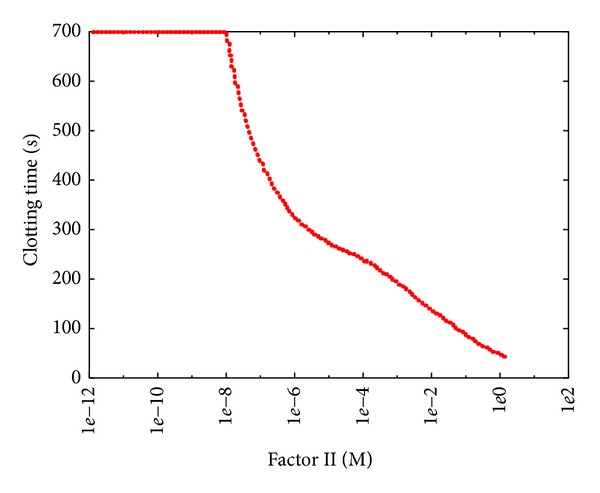
Clotting time at different initial concentrations of thrombin. The reference value is 1.40 · 10^−6^ M; the sweep range is [1.40 · 10^−12^, 1.40 · 10^0^].

The PSA-1D over factor II (Figure 11) shows a dramatic decrease of the CT as the initial concentration of this factor increases (with respect to the average physiological level). This behavior resembles the effects of hypercoagulability (or thrombophilia), a disease caused by mutation G20210A in the prothrombin gene [53] that causes an increase of the prothrombin level (factor II) in the blood flow, resulting in an excessive formation of the active form of this factor, thus heightening venous thrombosis risks [54]. Hypercoagulability is usually treated with warfarin therapy, or with other anticoagulants with a similar effect. These drugs decrease the capacity of coagulation factors to become active, preventing the formation of unwanted thrombi.

On the other hand, when the initial concentration of factor II is low, we achieved the effects of prothrombin deficiency, a rare autosomal recessive disease that causes a tendency to severe bleeding [29, 55]. As shown in Figure 11, a concentration equal to 10% of the physiological value of factor II (i.e., 1.4 · 10^−6^ M) leads to clotting effects similar to severe haemophilia A.

### 3.2. PSA-2D of the BCC Model

We present here the results of the PSA-2D on the BCC model, where couples of parameters were varied to analyze the possible effects arising from the combined perturbation of their values.

#### 3.2.1. Reactions *r*_27_ and *r*_58_

**Figure 12:**
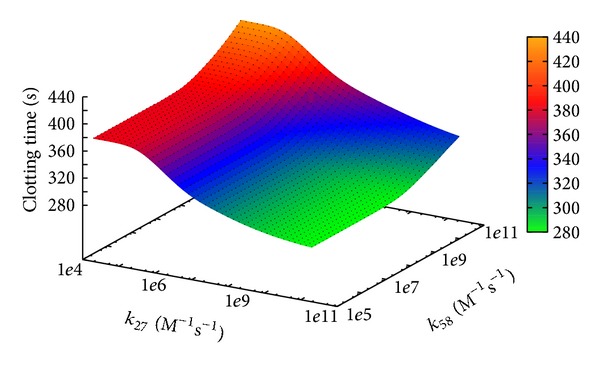
Clotting time according to PSA-2D over reaction constants *k*_27_ and *k*_58_. The sweep ranges are [2 · 10^4^, 2 · 10^10^] and [1 · 10^5^, 2 · 10^11^], respectively.

Figure 12 shows the effect of the simultaneous variation of constants *k*_27_ and *k*_58_ (over the same sweep ranges considered in the two PSA-1D, Section 3.1). This result remarks that reaction *r*_27_, involved in the common pathway, has a stronger influence on the BCC, and that there is a synergic interplay between these two reactions. In particular, when the value of *k*_27_ is low and the value of *k*_58_ is high, the CT is higher than the values achieved when only a single constant is changed, because in this condition both the intrinsic and the common pathways are simultaneously inhibited.

#### 3.2.2. Factor VIII, Factor IX, and Tissue Factor

In the last two PSA-2D we varied the initial concentration of factor VIII and Tissue Factor and the initial concentration of factor IX and Tissue Factor, respectively.

The initial concentrations of factors VIII and IX were varied over four orders of magnitude, using their physiological values as upper limit for the sweep ranges; the concentration of Tissue Factor was varied over four orders of magnitude, two above and two below its reference value (see Table 2). The rationale behind this choice is to observe how the BCC model, in conditions corresponding to different states of haemophilia (obtained by decreasing the concentrations of factors VIII and IX), behaves with different initial concentrations of the Tissue Factor, which is the upstream factor of the extrinsic pathway, that is, the most important element of the BCC.

**Figure 13:**
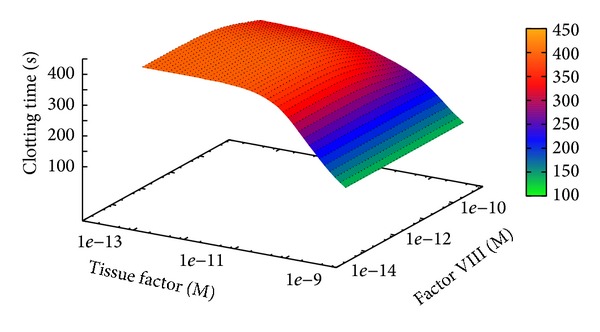
Clotting time at different initial concentrations of Tissue Factor and factor VIII. The sweep ranges are [5.00 · 10^−14^, 5.00 · 10^−10^] and [7.00 · 10^−14^, 5.00 · 10^−10^], respectively.

**Figure 14:**
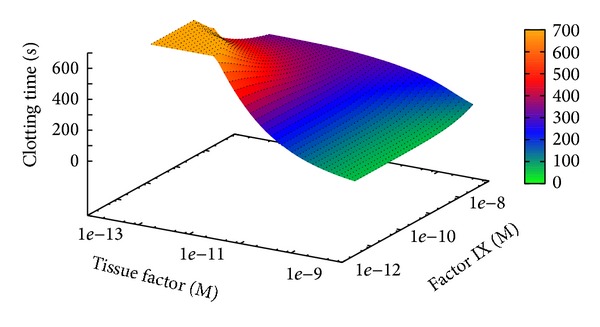
Clotting time at different initial concentrations of Tissue Factor and factor IX. The sweep ranges are [5.00 · 10^−14^, 5.00 · 10^−10^] and [9.00 · 10^−12^, 9.00 · 10^−8^], respectively.

The results of these PSA-2D show that, with respect to the condition of haemophilia B, in the case of haemophilia A the amount of Tissue Factor (below its reference value) has a negligible influence on the CT, as indicated by the presence of a plateau in Figure 13; on the contrary, concerning haemophilia B, a deficiency of Tissue Factor leads to an increase of the CT, especially when factor IX is present in low concentrations (Figure 14).

The different results achieved in the two PSA-2D are due to the presence, in the BCC model, of a direct interaction between Tissue Factor and factor IX by means of the TF-fVIIa complex (see reactions *r*_13_,…, *r*_15_ in Table 1). Indeed, the lack of Tissue Factor directly affects the concentration of active factor IX, which results in a strong alteration of the CT with respect to physiological conditions.

### 3.3. CPU versus GPU Performance Comparison

In order to show the relevant speedup achieved by coagSODA, we present here the comparison of the computational effort required by GPU and CPU for the simulation of the BCC model. The performances of our GPU simulator were compared with those obtained using the LSODA algorithm implemented in the software COPASI [56], executing on the CPU the same set of simulations that were run on the GPU. COPASI is single-threaded and does not exploit the physical and logical cores of the CPU; therefore, it represents a good benchmark as a single-node CPU-bound simulator of biological systems.

In all simulations, executed on both GPU and CPU, we stored 100 samples, uniformly distributed in the time interval considered for each simulation, that is, [0, 700] seconds, of the dynamics of all chemical species involved in the BCC model. The settings for the LSODA algorithm were the following: relative error 1 · 10^−7^, absolute error 1 · 10^−13^, and maximum number of internal steps set to 20000.

#### 3.3.1. Benchmark Details

The GPU used for the simulations is a Nvidia Tesla K20c, equipped with 2496 cores organized in 13 streaming multiprocessors, GPU clock 706 MHz, and 5 GB of DDR5 RAM. In all tests, coagSODA was compiled and executed with version 5.5 of CUDA libraries. Even though this GPU has compute capability 3.5 and is based on the GK110 Kepler architecture, currently coagSODA does not exploit any of the new features (e.g., Dynamic Parallelism, Hyper-Q, Remote DMA) with the exception of the new ISA encoding, which allows threads to exploit an increased number of registers (255 instead of 63), reducing register spilling into global memory and increasing performances [57]. Moreover, as described in Section 2.2, the Kepler architecture offers the possibility to reconfigure the 64KB on-chip cache, balancing between L1 cache and shared memory. Since coagSODA exploits the shared memory to reduce the latencies during the access to the concentration values of the BCC model, we opted for the following configuration: 48 KB for the shared memory, 16 KB for the L1 cache.

The performance of the GPU was compared against a personal computer equipped with a dual-core CPU Intel Core i5, frequency 2.3 GHz, 4 GB of DDR3 RAM, running the operating system Mac OS X Lion 10.7.5. The software used as a coagSODA-equivalent single-threaded CPU implementation is COPASI version 4.8 (build 35).

A direct comparison of the performances between these two different architectures and implementations is not an easy task, since CPUs are optimized for single-thread execution and exploit a large number of optimizations (e.g., higher clock frequency, instruction caching, pipelining, out of order execution, and branch prediction), whereas GPUs are optimized for graphics processing and parallel execution of identical code, relying only on multilevel data caching. For this reason, in this paper we propose an empirical comparison of the performances based on the running times of identical batches of simulations of the BCC model.

#### 3.3.2. Benchmark Results

**Figure 15:**
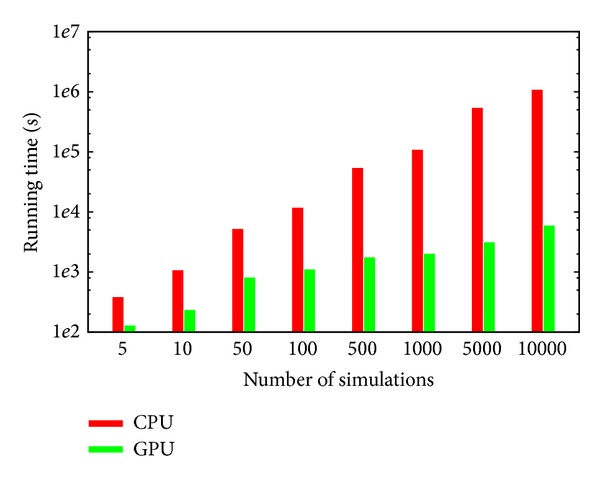
Comparison of the computational time required to execute an increasing number of simulations of the BCC model on CPU (Intel Core i5, 2.3 GHz) and GPU (Nvidia Tesla K20c, GPU clock 706 MHz). The computational time is expressed in seconds. The time values related to 1000, 5000, and 10000 simulations on the CPU were estimated by regression (see also Table 3).

In Figure 15 we show the comparison of the running times required to run several batches of simulations, executed to carry out the PSA-1D over the reaction constant *k*_27_. The choice of comparing the performances of the GPU and CPU implementations by executing batches of simulations that are related to a PSA, instead of running *n* independent but identical simulations (i.e., all characterized by the same parameterization of the model), is due to the fact that these results represent a more realistic scenario in the computational analysis of biological systems, whereby it is useful to investigate large search spaces of parameters, corresponding to different perturbed conditions of the model [26]. Moreover, for the evaluation of the running time, the execution of a batch of *n* identical deterministic simulations would be futile. The figure clearly shows that coagSODA always performs better than the CPU counterpart. In particular, while the CPU performance increases linearly with the number of simulations, the running times are strongly reduced on the GPU; in this case, the overall running time roughly corresponds to the time required for the execution of the slowest simulations. This is due to the fact that different parameterizations may require different time steps for LSODA to converge, and the execution of a block terminates as long as all the threads it contains terminate. In turn, the execution of a kernel terminates when all blocks terminate; for this reason, a single simulation, whose dynamics requires more steps than the others, may affect the overall running time.

In Table 3 we report the running times of all batches of simulations, along with the speedup achieved on the GPU. In particular, these results highlight that the advantage of exploiting the GPUs for the simulation of the BCC model becomes more evident as the number of simulations increases, with a 181× speedup when a PSA with 10000 different parameterizations is executed. Therefore, the GPU accelerated analysis of the BCC model with coagSODA represents a novel, relevant computational means to investigate the behavior of this complex biological system under nonphysiological conditions and could be exploited to efficiently determine the response of the BCC to different therapeutical drugs.

## 4. Conclusions

Thanks to their high-performance computing capabilities and the very low costs, GPUs nowadays represent a suitable technology for the development and the application of parallel computational methods for* in silico* analysis of complex biological systems. However, the implementation of efficient computational tools able to fully exploit the large potentiality of GPUs is still challenging, since good programming skills are required to implement GPU-based algorithmic methods, and to handle specific features as an optimal usage of memory or the communication bandwidth between GPU and CPU. Moreover, algorithms cannot be directly ported on the GPU because of the limited programming capabilities allowed by GPU kernels; as a matter of fact, they need to be redesigned to meet the requirements of the underlying SIMD architecture.

In this work we presented coagSODA, a GPU-powered simulator specifically developed to carry out fast parallel simulations of the BCC model. coagSODA was designed to offer a black-box solution usable by any final user in an easy way. It relies on cupSODA [24], a numerical integrator for ODEs that we previously implemented for the GPU architecture, based on the LSODA algorithm and capable of automatically translating a reaction-based model into a set of coupled ODEs. In addition to mass-action kinetics, coagSODA implements specific functions to compute the kinetics of Hill function based reactions, such as those involved in the platelets activity of the BCC model. coagSODA exploits the massive parallel capabilities of modern GPUs, and our results demonstrated that it can achieve a relevant reduction of the computational time required to execute many concurrent and independent simulations of the BCC dynamics. The mutual independence of the simulations allows fully exploiting the underlying SIMD architecture; moreover, coagSODA benefits from an additional speedup, thanks to our choice of storing the state of the system into the low-latency shared memory (a solution that was already implemented in cupSODA). Since the BCC model is large (96 reactions, 71 chemical species), a large amount of shared memory was assigned to each thread, strongly reducing the theoretical occupancy of the GPU, that is, the ratio of active warps with respect to the maximum number of warps supported by each streaming multiprocessor of the GPU. However, the results of the analysis presented in this work, performed on the BCC model, show that coagSODA achieves a relevant boost with respect to a reference CPU implementation. For instance, in the case of 10000 simulations, we achieved a noticeable 181× speedup. Interestingly, the performances of coagSODA are better than COPASI even for small batches of simulations. These results indicate that, for biological models consisting in many reactions and many species, our GPU implementation of LSODA becomes more profitable than the CPU counterpart as the number of concurrent simulations increases, making it suitable especially when performing demanding computational analysis such as, for example, parameter sweep, parameter estimation, or sensitivity analysis.

As a test case, in this paper we presented several parameter sweep analyses over reaction constants and initial concentrations of factors involved in the BCC model. Other computational analyses on mathematical models of this pathway were previously presented. For instance, in [58], a parameter estimation of the reaction constants of different models involving the activation of factor X, factor V, prothrombin, and the inactivation of factors was performed. The aim of this analysis was to discriminate between different models to unravel the mechanisms on the basis of the BCC. Similar analyses were executed on a model describing thrombin generation in plasma, since the reaction mechanism, the reaction constants, and initial concentrations were unknown [59]. In [34], a sensitivity analysis of a model consisting of 44 species over 34 chemical species was presented; reaction constants were varied between 10% and 1000% of their reference value, to the aim of identifying the most influential factors of the BCC.

All these analyses were performed by means of sequential simulations of the models under investigation; therefore, in general, only small batches of simulations could be run (for instance, in [34] only 836 simulations were executed to execute the sensitivity analysis of the model). On the other hand, in this paper we presented the results of different PSA, efficiently executed by means of coagSODA, overall providing useful information regarding unknown parameters and interesting insights into the functioning of the BCC. A thorough sensitivity analysis of the whole BCC model, consisting in around 5 · 10^5^ simulations, is currently in progress on GPUs by our group, exploiting the great computational efficiency of coagSODA.

The results of our computational analyses should be now validated by means of specifically designed laboratory experiments. In particular, we identified a plausible value for the constant of the reaction describing the autoactivation of factor XII (since no reference values can be found in literature); moreover, the results of the PSA over factor IX suggest that a deficiency of this factor is not enough to cause severe bleeding disorders as haemophilia B, but an alteration of other factors seems to be necessary for the occurrence of such condition (e.g., the lack of Tissue Factor, as suggested by the PSA-2D over Tissue Factor and factor IX). Finally, the PSA over factors VIII and IX showed, in a situation characterized by very high concentrations of these factors, a counterintuitive behavior in which the clotting time is increased with respect to the value obtained in physiological conditions. Indeed, it would be interesting to design* ad hoc* laboratory experiments to verify if the BCC model is actually predictive in such extreme situations.

The coagSODA software and the SBML version of the BCC model used in this work are available from the authors upon request.

## Figures and Tables

**Table 1:** Reaction-based model of the blood coagulation cascade (reduced version of the “Platelet-Plasma” model described in [30]). The model consists in 96 reactions among 71 molecular species. With the exception of reaction *r*_44_ (see Section 3.1), the values of all reaction constants were taken from [30].

*n*	Reactants	Products	Constant (*k*)
*r*_1_	TF + fVII	TF-fVII	3.20 · 10^6^ M^−1^ s^−1^
*r*_2_	TF-fVII	TF + fVII	3.10 · 10^−2^ s^−1^
*r*_3_	TF + fVIIa	TF-fVIIa	2.30 · 10^7^ M^−1^ s^−1^
*r*_4_	TF-fVIIa	TF + fVIIa	3.10 · 10^−5^ s^−1^
*r*_5_	TF-fVIIa + fVII	TF-fVIIa + fVIIa	4.40 · 10^5^ M^−1^ s^−1^
*r*_6_	fXa + fVII	fXa + fVIIa	1.30 · 10^7^ M^−1^ s^−1^
*r*_7_	fIIa + fVII	fIIa + fVIIa	2.30 · 10^4^ M^−1^ s^−1^
*r*_8_	TF-fVIIa + fX	TF-fVIIa-fX	2.50 · 10^7^ M^−1^ s^−1^
*r*_9_	TF-fVIIa-fX	TF-fVIIa + fX	1.05 · 10^−2^ s^−1^
*r*_10_	TF-fVIIa-fX	TF-fVIIa-fXa	6.00 s^−1^
*r*_11_	TF-fVIIa-fXa	TF-fVIIa + fXa	19.00 s^−1^
*r*_12_	TF-fVIIa + fXa	TF-fVIIa-fXa	2.20 · 10^7^ M^−1^ s^−1^
*r*_13_	TF-fVIIa + fIX	TF-fVIIa-fIX	1.00 · 10^7^ M^−1^ s^−1^
*r*_14_	TF-fVIIa-fIX	TF-fVIIa + fIX	2.40 s^−1^
*r*_15_	TF-fVIIa-fIX	TF-fVIIa + fIXa	1.80 s^−1^
*r*_16_	fII + fXa	fIIa + fXa	7.50 · 10^3^ M^−1^ s^−1^
*r*_17_	fIIa + fVIII	fIIa + fVIIIa	2.00 · 10^7^ M^−1^ s^−1^
*r*_18_	fVIIIa + fIXa	fIXa-fVIIIa	1.00 · 10^7^ M^−1^ s^−1^
*r*_19_	fIXa-fVIIIa	fVIIIa + fIXa	1.00 · 10^−4^ s^−1^
*r*_20_	fIXa-fVIIIa + fX	fIXa-fVIIIa-fX	1.00 · 10^8^ M^−1^ s^−1^
*r*_21_	fIXa-fVIIIa-fX	fIXa-fVIIIa + fX	1.00 · 10^−5^ s^−1^
*r*_22_	fIXa-fVIIIa-fX	fIXa-fVIIIa + fXa	8.20 s^−1^
*r*_23_	fVIIIa	fVIIIa_1_-L + fVIIIa_2_	6.00 · 10^−5^ s^−1^
*r*_24_	fVIIIa_1_-L + fVIIIa_2_	fVIIIa	2.20 · 10^4^ M^−1^ s^−1^
*r*_25_	fIXa-fVIIIa-fX	fVIIIa_1_-L + fVIIIa_2_ + fX + fIXa	1.00 · 10^−3^ s^−1^
*r*_26_	fIXa-fVIIIa	fVIIIa_1_-L + fVIIIa_2_ + fIXa	1.00 · 10^−3^ s^−1^
*r*_27_	fIIa + fV	fIIa + fVa	2.00 · 10^7^ M^−1^ s^−1^
*r*_28_	fXa + fVa	fXa-fVa	4.00 · 10^8^ M^−1^ s^−1^
*r*_29_	fXa-fVa	fXa + fVa	0.2 s^−1^
*r*_30_	fXa-fVa + fII	fXa-fVa-fII	1.00 · 10^8^ M^−1^ s^−1^
*r*_31_	fXa-fVa-fII	fXa-fVa + fII	103.00 s^−1^
*r*_32_	fXa-fVa-fII	fXa-fVa + fmIIa	63.50 s^−1^
*r*_33_	fXa-fVa + fmIIa	fXa-fVa + fIIa	1.50 · 10^7^ M^−1^ s^−1^
*r*_34_	fXa + TFPI	fXa-TFPI	9.00 · 10^5^ M^−1^ s^−1^
*r*_35_	fXa-TFPI	fXa + TFPI	3.60 · 10^−4^ s^−1^
*r*_36_	TF-fVIIa-fXa + TFPI	TF-fVIIa-fXa-TFPI	3.20 · 10^8^ M^−1^ s^−1^
*r*_37_	TF-fVIIa-fXa-TFPI	TF-fVIIa-fXa + TFPI	1.10 · 10^−2^ s^−1^
*r*_38_	TF-fVIIa + fXa-TFPI	TF-fVIIa-fXa-TFPI	5.00 · 10^7^ M^−1^ s^−1^
*r*_39_	fXa + ATIII	fXa-ATIII	1.50 · 10^3^ M^−1^ s^−1^
*r*_40_	fmIIa + ATIII	fmIIa-ATIII	7.10 · 10^3^ M^−1^ s^−1^
*r*_41_	fIXa + ATIII	fIXa-ATIII	4.90 · 10^2^ M^−1^ s^−1^
*r*_42_	fIIa + ATIII	fIIa-ATIII	7.10 · 10^3^ M^−1^ s^−1^
*r*_43_	TF-fVIIa + ATIII	TF-fVIIa-ATIII	2.30 · 10^2^ M^−1^ s^−1^
*r*_44_	fXII	fXIIa	5.00 · 10^−4^ s^−1^
*r*_45_	fXIIa + fXII	fXIIa-fXII	1.00 · 10^8^ M^−1^ s^−1^
*r*_46_	fXIIa-fXII	fXIIa + fXII	750.00 s^−1^
*r*_47_	fXIIa-fXII	fXIIa + fXIIa	3.30 · 10^−2^ s^−1^
*r*_48_	fXIIa + PKal	fXIIa-PKal	1.00 · 10^8^ M^−1^ s^−1^
*r*_49_	fXIIa-PKal	fXIIa + PKal	3.60 · 10^3^ s^−1^
*r*_50_	fXIIa-PKal	fXIIa + Kal	40.00 s^−1^
*r*_51_	fXII + Kal	fXII-Kal	1.00 · 10^8^ M^−1^ s^−1^
*r*_52_	fXII-Kal	fXII + Kal	45.30 s^−1^
*r*_53_	fXII-Kal	fXIIa + Kal	5.70 s^−1^
*r*_54_	PKal + Kal	Kal + Kal	2.70 · 10^4^ M^−1^ s^−1^
*r*_55_	Kal	Kal_*i*_	1.10 · 10^−2^ s^−1^
*r*_56_	fXIIa + C1inh	fXIIa-C1inh	3.60 · 10^3^ M^−1^ s^−1^
*r*_57_	fXIIa + ATIII	fXIIa-ATIII	21.60 M^−1^ s^−1^
*r*_58_	fXI + fIIa	fXI-fIIa	1.00 · 10^8^ M^−1^ s^−1^
*r*_59_	fXI-fIIa	fXI + fIIa	5.00 s^−1^
*r*_60_	fXI-fIIa	fXIa + fIIa	1.30 · 10^−4^ s^−1^
*r*_61_	fXIIa + fXI	fXIIa-fXI	1.00 · 10^8^ M^−1^ s^−1^
*r*_62_	fXIIa-fXI	fXIIa + fXI	200 s^−1^
*r*_63_	fXIIa-fXI	fXIIa + fXIa	5.70 · 10^−4^ s^−1^
*r*_64_	fXIa + fXI	fXIa + fXIa	3.19 · 10^6^ M^−1^ s^−1^
*r*_65_	fXIa + ATIII	fXIa-ATIII	3.20 · 10^2^ M^−1^ s^−1^
*r*_66_	fXIa + C1inh	fXIa-C1inh	1.80 · 10^3^ M^−1^ s^−1^
*r*_67_	fXIa + A1AT	fXIa-A1AT	1.00 · 10^2^ M^−1^ s^−1^
*r*_68_	fXIa + A2AP	fXIa-A2AP	4.3 · 10^3^ M^−1^ s^−1^
*r*_69_	fXIa + fIX	fXIa-fIX	1.00 · 10^8^ M^−1^ s^−1^
*r*_70_	fXIa-fIX	fXIa + fIX	41.00 s^−1^
*r*_71_	fXIa-fIX	fXIa + fIXa	7.70 s^−1^
*r*_72_	fIXa + fX	fIXa-fX	1.00 · 10^8^ M^−1^ s^−1^
*r*_73_	fIXa-fX	fIXa + fX	0.64 s^−1^
*r*_74_	fIXa-fX	fIXa + fXa	7.00 · 10^−4^ s^−1^
*r*_75_	fXa + fVIII	fXa-fVIII	1.00 · 10^8^ M^−1^ s^−1^
*r*_76_	fXa-fVIII	fXa + fVIII	2.10 s^−1^
*r*_77_	fXa-fVIII	fXa + fVIIIa	0.023 s^−1^
*r*_78_	fVIIa + fIX	fVIIa-fIX	1.00 · 10^8^ M^−1^ s^−1^
*r*_79_	fVIIa-fIX	fVIIa + fIX	0.90 s^−1^
*r*_80_	fVIIa-fIX	fVIIa + fIXa	3.60 · 10^−5^ s^−1^
*r*_81_	fVIIa + fX	fVIIa-fX	1.00 · 10^8^ M^−1^ s^−1^
*r*_82_	fVIIa-fX	fVIIa + fX	210.00 s^−1^
*r*_83_	fVIIa-fX	fVIIa + fXa	1.60 · 10^−6^ s^−1^
*r*_84_	Fbg + fIIa	Fbg-fIIa	1.00 · 10^8^ M^−1^ s^−1^
*r*_85_	Fbg-fIIa	Fbg + fIIa	636.00 s^−1^
*r*_86_	Fbg-fIIa	Fbn1 + fIIa + FPA	84.00 s^−1^
*r*_87_	Fbn1 + fIIa	Fbn1-fIIa	1.00 · 10^8^ M^−1^ s^−1^
*r*_88_	Fbn1-fIIa	Fbn1 + fIIa	742.60 s^−1^
*r*_89_	Fbn1-fIIa	Fbn2 + fIIa + FPB	7.40 s^−1^
*r*_90_	Fbn1 + Fbn1	(Fbn1)_2_	1.00 · 10^6^ M^−1^ s^−1^
*r*_91_	(Fbn1)_2_	2Fbn1	6.40 · 10^−2^ s^−1^
*r*_92_	(Fbn1)_2_ + fIIa	(Fbn1)_2_-fIIa	1.00 · 10^8^ M^−1^ s^−1^
*r*_93_	(Fbn1)_2_-fIIa	(Fbn1)_2_ + fIIa	701.00 s^−1^
*r*_94_	(Fbn1)_2_-fIIa	(Fbn2)_2_ + fIIa + FPB	49.00 s^−1^
*r*_95_	Fbn2 + fIIa	Fbn2-fIIa	1.00 · 10^8^ M^−1^ s^−1^
*r*_96_	Fbn2-fIIa	Fbn2 + fIIa	1.00 · 10^3^ s^−1^

**Table 2:** Initial concentrations of molecular species in the blood coagulation cascade model (values taken from [30]).

Species	Symbol	Concentration (M)
α1-Antitrypsin	A1AT	4.50 · 10^−5^
α2-Antiplasmin	A2AP	1.00 · 10^−6^
Antithrombin III	ATIII	3.40 · 10^−6^
C1-inhibitor	C1inh	2.50 · 10^−6^
Fibrinogen	Fbg	9.00 · 10^−6^
Factor II	fII	1.40 · 10^−6^
Factor V	fV	2.00 · 10^−8^
Factor VII	fVII	1.00 · 10^−8^
Active factor VII	fVIIa	1.00 · 10^−10^
Factor VIII	fVIII	7.00 · 10^−10^
Factor IX	fIX	9.00 · 10^−8^
Factor X	fX	1.6 · 10^−7^
Factor XI	fXI	3.10 · 10^−8^
Factor XII	fXII	3.40 · 10^−7^
Prekallikrein	Pkal	4.50 · 10^−7^
Tissue Factor	TF	5.00 · 10^−12^
Tissue Factor Pathway Inhibitor	TFPI	2.50 · 10^−9^

**Table 3:** CPU versus GPU performance comparison.

Number of simulations	CPU time (sec)	GPU time (sec)	Speedup
5	380.8	127.8	2.98×
10	1060.4	232.9	4.56×
50	5170.4	805.8	6.42×
100	11652.8	1097.7	10.61×
500	53605.8	1739.4	30.81×
1000	107358.2∗	1998.2	53.73×
5000	536350.7∗	3096.0	173.2×
10000	1072497.5∗	5895.1	181.9×

*Values estimated by regression.
